# Incorporation and effects of punicic acid on muscle and adipose tissues of rats

**DOI:** 10.1186/s12944-016-0214-7

**Published:** 2016-02-27

**Authors:** Illana Louise Pereira de Melo, Ana Mara de Oliveira e Silva, Eliane Bonifácio Teixeira de Carvalho, Luciana Tedesco Yoshime, José Augusto Gasparotto Sattler, Jorge Mancini-Filho

**Affiliations:** Department of Food and Experimental Nutrition, Laboratory of Lipids, Faculty of Pharmaceutical Sciences, São Paulo, University of São Paulo, Av. Prof. Lineu Prestes, 580 – Bloco 14, CEP: 05508-900 São Paulo, Brazil; Federal University of Sergipe, Biological Science and Health Center, Aracaju, Sergipe Brazil

**Keywords:** Conjugated linolenic acid, Punicic acid, Fatty acid profile, Oxidative status

## Abstract

**Background:**

This study evaluated the effect of pomegranate seed oil (PSO) supplementation, rich in punicic acid (55 %/C18:3-9*c*,11 *t*,13*c*/CLNA), on the lipid profile and on the biochemical and oxidative parameters in the gastrocnemius muscle and adipose tissues of healthy rats. Linseed oil (LO), rich in linolenic acid (52 %/C18:3-9*c*12*c*15*c*/LNA) was used for comparison.

**Methods:**

Male Wistar rats (*n* = 56) were distributed in seven groups: control (water); LNA 1 %, 2 % and 4 % (treated with LO); CLNA 1 %, 2 % and 4 % (treated with PSO), po for 40 days. The percentages were compared to the daily feed intake. Fatty acid profile were performed by gas chromatography, antioxidant enzymes activity by spectrophotometer and the adipocytes were isolated by collagenase tissue digestion. Analysis of variance (ANOVA) was applied to check for differences between the groups (control, LNAs and CLNAs) and principal component analysis (PCA) was used to project the groups in the factor-place (PC1 vs PC2) based on the biochemical responses assessed in the study.

**Results:**

The fatty acids profile of tissues showed that the LNA percentages were higher in the animals that were fed LO. However, PA was only detected in the adipose tissues. Conjugated linoleic acid (CLA) was present in all the tissues of the animals supplemented with PSO, in a dose dependent manner, and 9*c*11*t*-CLA was the predominant isomer. Nevertheless there were no changes in the total weight gain of the animals, the weights of the tissues, and the oxidative stress parameters in the muscle. In addition, there was an increase in the size of the epididymal fat cells in the groups treated with PSO. Principal component analysis (PCA) showed that the CLNAs groups were arranged separately with a cumulative variance of 68.47 %.

**Conclusions:**

The results show that PSO can be used as a source of CLAs but that it does not cause changes in body modulation and does not interfere in the antioxidant activity of healthy rats.

## Highlights

- Effects of pomegranate seed oil supplementation were investigated.
- Lipid profile and biochemical parameters in rat’s tissues are analyzed.
- Pomegranate seed oil supplementation did not reduced adipose tissue.
- Punicic acid was incorporated in adipose tissue but not in muscle.
- Punicic acid was metabolized to conjugated linoleic acid.

## Background

Since its discovery, the conjugated linoleic acids (CLAs) have been extensively investigated with respect to its beneficial effects to the body, with studies focused mainly on their anti-adipogenic activity. However, the results so far are inconclusive and controversial. Another type of conjugated, conjugated linolenic acid (CLNAs), has also been drawing attention recently and has been the subject of research to evaluate its effects on the body. Because they are metabolized to CLAs form in several animal models, studies are suggesting that they have the same effects of CLAs. In this line, the objective of this study was to evaluate the supplementation effect with pomegranate seed oil (PSO), rich in Punicic acid (CLNA) on the lipid profile and on the biochemical and oxidative parameters in the muscle and adipose tissues of healthy rats

## Introduction

Conjugated fatty acids (CFAs) is the general term for a group of positional and geometric isomers of polyunsaturated fatty acids with conjugated double bonds [1]. Interest in the health benefits of CFAs is increasing because they have been shown to possess pharmacological activities which are relevant to the prevention and treatment of atherosclerosis, obesity, cancer and hypertension [2, 3].

There are CFAs of linoleic acid and α-linolenic acid. Conjugated linoleic acids (CLAs) comprise a group of positional and geometrical isomers of octadecadienoic acid (18:2 ω–6), in which the two double bonds are conjugated. CLAs naturally occur in foods from ruminants and in milk and dairy products in very small amounts [4]. Conjugated α-linolenic acids (CLNAs) have three double bonds conjugated together. Unlike CLA, CLNA isomers are present at much higher levels in certain seed oils [5].

There are many studies evaluating the beneficial effects of CLAs to the body, especially in some experimental animal models (mice, rats, hamsters, pigs). The main focus of these studies is the ability of CLAs to reduce adipose tissue in several animal models, as well as affecting muscle mass [6, 7]. Another factor that has drawn attention is that it has been shown that CLAs have in vitro and in vivo antioxidant activity, and this could be a possible explanation for their beneficial health effects [8, 9]. However, some results are controversial and even show other adverse effects. In addition, the effects in humans are still questionable, and the biochemical mechanisms through which CLAs mediate their potential effects could produce undesirable metabolic alterations [3, 10]. Furthermore, differences have been observed in the effects/benefits of CLAs, both in association and between their individual isomers, which may partly explain some of the inconsistent results obtained experimentally [7, 10, 11].

Although the information on the effects of CLNAs is even more limited compared to CLAs, and does not provide a consensus in the literature regarding their effects on animal and human organisms, some studies have demonstrated that CLNAs have a cytotoxic effect on cultured human tumor cells [12–14], that they inhibit carcinogenesis [15–17], and that they alter lipid metabolism in animals [18–21]. It has been found that different CLNA isomers exert different bioactivities [22] but their mechanisms of action are unclear. Furthermore, studies have shown that CLNAs could be metabolized into CLA in vivo [23–25], suggesting that the health benefits may be due to the CLA. These researchers suggest that CLnAs may be metabolized to CLAs via a Δ13 saturation reaction catalyzed by a NADPH (nicotinamide adenine dinucleotide phosphate) dependent enzyme, which is either a novel enzyme capable of recognizing conjugated trienoic acid or the enzyme active in the leukotriene B4 reductive pathway. The incorporation and metabolism of orally administered punicic acid (PA), one isomer of CLNA, in rat tissues and plasma were studied over a 24-h period by Yuan et al. [25]. The results show that PA was incorporated and metabolized to 9*c*,11 *t* CLA in rat plasma, liver, kidney, heart, brain, and adipose tissue. Therefore natural resources containing CLNA could be a potential dietary source of CLA, following PA metabolism.

PA (C18:3-9*c*,11*t*,13*c*), which is also known as trichosanic acid, is an isomer of CLNAs that has aroused great interest in recent years. It is found in high amounts (> 50 %) in pomegranate seed oil (PSO) [26]. For this reason, PSO has been increasingly investigated as a potent functional food and/or nutraceutical ingredient in foods [27]. According to Koba et al. [28], because pomegranate abounds in PA its seed oil represents a suitable source for the investigation of the physiological roles of this CLNA isomer.

Given the limited and often contradictory information that exists about this issue, further studies regarding the function of CLNAs in animal organisms, as well as their single isomers, is required [26]. Furthermore, most studies assess the effects of CLNAs in specific physiological conditions such as cancer and other diseases, the physiological effects and mechanisms of action of punicic acid in healthy body have not been well characterized. We hypothesized that punicic acid present in the PSO it can be incorporated in the form of CLA in the animal tissues and present the effects in body composition modulation (reducing body fat and increased lean mass), lipid profile and antioxidant activity in this tissues, which are attributed to supplementation with CLAs isomers. Hence the aim of this study was to evaluate the effect of supplementation with pomegranate seed oil, rich in punicic acid, on the lipid profile, and also its influence on the biochemical and oxidative parameters in the muscle and adipose tissue of healthy rats.

## Results and discussion

Table 1 shows the results in relation to weight gain, feed and calorie intake, feed efficiency coefficient (FEC) and tissue weight. Supplementation with PSO and LO significantly reduced total food intake in the groups supplemented with a dose of 4 % but there was no significant difference between the groups regarding the total weight gain of the animals. This result can be explained by the amount of calories consumed by the animals. Adding together the calories from the consumption of commercial feed that was offered *ad libitum* (Kcal/gram of feed) to the calories in the amount of oil that was offered, no differences were observed between the groups. The results of the gastrocnemius muscle weight, retroperitoneal adipose tissue and the epididymal adipose tissue are presented as a percentage relative to the total body weight of the animals. These results show that supplementation with both LO and with PSO in all the concentrations employed, did not statistically affect the weight of these tissues.

**Table 1:** Body weight and feed intake of rats supplemented with different oils

Parameters	Control	LNA 1 %	LNA 2 %	LNA 4 %	CLNA 1 %	CLNA 2 %	CLNA 4 %
Initial weight (g)	110 ± 10	107 ± 15	111 ± 10	113 ± 11	114 ± 5	112 ± 7	114 ± 10
Weight gain (g)	211 ± 13	212 ± 14	209 ± 23	205 ± 22	209 ± 17	210 ± 16	213 ± 23
Total feed intake (g)	1023 ± 40^a^	968 ± 46^abc^	971 ± 7^abc^	884 ± 2^c^	1035 ± 26^a^	1000 ± 73^ab^	926 ± 11^bc^
FEC	0.21	0.22	0.22	0.23	0.20	0.21	0.23
Calorie intake (Kcal)	2985 ± 115	2912 ± 134	3006 ± 20	2897 ± 6	3111 ± 76	3093 ± 214	3033 ± 34
Muscle (%w/w)	1.96 ± 0.44	1.75 ± 0.19	1.80 ± 0.11	1.84 ± 0.19	1.83 ± 0.29	1.73 ± 0.27	1.73 ± 0.26
Retroperitoneal (%w/w)	2.32 ± 0.57	2.55 ± 0.60	3.02 ± 1.02	2.77 ± 0.41	2.95 ± 0.89	2.41 ± 0.64	2.57 ± 0.74
Epididymal (%w/w)	2.76 ± 0.69	3.15 ± 0.61	3.14 ± 0.78	3.11 ± 0.57	3.39 ± 0.78	2.82 ± 0.65	3.29 ± 0.71

Results expressed as mean ± standard deviation. Different letters in the same row are statistically different from each other (*p* < 0.05)

(*%w/w*) percentage of tissue weight compared to total body weight, *FEC* feed efficiency coefficient

Conjugated fatty acids are commonly reported as a reductor of adipose tissue while increasing body lean mass [3, 20, 25, 29] and therefore, several studies have been conducted aiming to change the body weight of the animals that consumed CLNAs. However, studies have reported conflicting results regarding the role of CLNAs in weight gain and body composition. Koba et al. [30] evaluated how CLNAs affected body fat in rats and they found that feeding CLNAs resulted in a reduction in the adipose tissue weight. In contrast, supplementation with 1 % CLNAs (PA and/or α-ESA = α-eleostearic acid C18:3-9*c*,11 *t*,13 *t*) for six weeks did not significantly affect food intake, body weight or the tissues in mice [25, 31]. Yamasaki et al. [19] reported that body weight and adipose tissue were not affected in mice fed with experimental diets containing 0.12 and 1.2 % of PSO that was rich in PA, for three weeks. Arao et al. [32] showed that consumption of a diet supplemented with 9 % safflower oil and 1 % PSO for two weeks did not affect the weight of abdominal white adipose tissue in Otsuka Long-Evans Tokushima Fatty rats (OLETF- spontaneously hyperglycemic rats with long-term diabetic complications). However, Koba et al. [30] showed that CLNAs decreased the weight of perirenal fat tissue to a greater extent, when compared to linoleic acid (LA), α-linolenic acid (LNA) and conjugated linoleic acid (CLA) in rats. The same group showed that supplementation with PSO decreased the weight of perirenal adipose tissue of mice in a dose-dependent manner after four weeks of feeding [20]. In contrast, Nemer [33] reported that mice fed with 2 % PA showed higher body weight gain and improved feed efficiency coefficient when compared to animals fed with 1 % PA and the control diet for 16 weeks. In addition, the animals fed with 1 % and 2 % of PA had reduced muscle weight (quadriceps) compared to the control group, but no difference was observed regarding the weight of the epididymal adipose tissue.

In the present study, the lipids obtained from the animal tissues supplemented with LO and PSO were extracted to analyze the fatty acid profile of the same. Therefore, it was possible to estimate the total lipid content of each tissue, as can be seen in Tables 2, 3 and 4, which show the percentage of fatty acids found in the lipids of the muscle, and the retroperitoneal and epididymal adipose tissues. Supplementation with PSO did not alter the lipid content of the muscle (gastrocnemius) and adipose (retroperitoneal and epididymal) tissues. The analysis of the composition of fatty acids in these tissues showed that, in general, the percentages of α-linolenic acid (LNA) were higher in the animals fed linseed oil (LO) in a dose-dependent manner. However, only traces of punicic acid (PA) were found in the muscle tissue of animals supplemented with PSO/CLNA. Nevertheless, it was possible to detect this fatty acid in the adipose tissue in percentages ranging from 2.13 to 4.99 % (retroperitoneal) and 2.13 to 4.83 % (epididymal). Interestingly, CLAs were present in all the tissues, in a dose-dependent manner in relation to what was provided. The 9*c*11*t*-CLA isomer was the predominant form and it was found in an even higher percentage than PA in the fat tissue (3.18 % to 9.58 %). In terms of the gastrocnemius muscle it was observed that there was also an increase in the C22:4 ω-6 in the groups receiving LO. However, this fatty acid was reduced in all the groups supplemented with LO and PSO when compared with the control group in the fatty tissues. In the epididymal adipose tissue it was also possible to observe a reduction in fatty acids C18:1ω-9, C18:2ω-6 and C22:6ω-3 in the CLNA-supplemented groups.

**Table 2:** Fatty acids (FAs) profile in gastrocnemius muscle tissue of rats supplemented with different oils

FAs (%)	Control	LNA 1 %	LNA 2 %	LNA 4 %	CLNA 1 %	CLNA 2 %	CLNA 4 %
C16:0	21.0 ± 0.6	20.1 ± 0.7	20.3 ± 0.8	20.6 ± 1.0	23.3 ± 2.2	23.1 ± 2.0	23.0 ± 2.8
C18:0	14.6 ± 2.4	15.0 ± 0.8	15.1 ± 1.8	17.5 ± 1.7	15.7 ± 1.8	16.0 ± 2.2	16.9 ± 2.6
C18:1*c* ω-9	11.2 ± 4.6	10.3 ± 0.2	9.7 ± 1.7	8.8 ± 1.5	12.1 ± 5.5	11.1 ± 5.3	10.0 ± 3.1
C18:2*c* ω-6	26.7 ± 1.4	27.6 ± 1.3	26.8 ± 1.5	25.0 ± 0.9	26.3 ± 3.6	26.1 ± 1.7	25.9 ± 2.4
C18:3*c* ω-3	1.0 ± 0.3^a^	2.4 ± 0.2^b^	3.7 ± 0.7^bc^	4.4 ± 1.2	0.9 ± 0.4^a^	1.0 ± 0.3^a^	0.9 ± 0.2^a^
CLA 9*c*11*t*					1.3 ± 0.4^a^	2.3 ± 0.6^ab^	3.4 ± 1.1^b^
CLA 10*c*12*c*					0.7 ± 0.4^a^	1.1 ± 0.3^ab^	1.6 ± 0.4^b^
C20:4 ω-6	15.2 ± 2.2	11.5 ± 0.3	10.9 ± 1.1	9.7 ± 1.1	10.0 ± 6.8	11.6 ± 3.1	11.2 ± 2.1
C22:4 ω-6	2.2 ± 0.2^a^	3.7 ± 0.2^b^	4.4 ± 0.3^bc^	5.4 ± 0.4	2.5 ± 0.4^a^	2.0 ± 0.4^a^	1.9 ± 0.2^a^
C22:6 ω-3	8.1 ± 1.6^ab^	9.3 ± 0.4^a^	9.0 ± 0.5^a^	8.5 ± 0.5^ac^	7.1 ± 1.8^ac^	5.7 ± 2.3^bc^	5.2 ± 0.6^b^
**TL (%)**	1.4 ± 0.1	1.5 ± 0.1	1.6 ± 0.3	1.4 ± 0.2	1.5 ± 0.3	1.3 ± 0.3	1.3 ± 0.3

Results expressed as mean ± standard deviation (*n* = 4) of the fatty acid percentages; different letters in the same row are statistically different from each other (*p* < 0.05)

*CLA* conjugated fatty acid (C18:2), *TL* total lipids

**Table 3:** Fatty acids (FAs) profile in retroperitoneal adipose tissue of rats supplemented with different oils

FAs (%)	Control	LNA 1 %	LNA 2 %	LNA 4 %	CLNA 1 %	CLNA 2 %	CLNA 4 %
C13:0	3.00 ± 0.62	2.74 ± 0.44	2.95 ± 0.43	2.79 ± 0.71	2.67 ± 0.35	2.75 ± 0.47	2.62 ± 0.40
C14:0	1.14 ± 0.04	0.93 ± 0.06	0.98 ± 0.11	0.95 ± 0.04	1.08 ± 0.09	1.01 ± 0.08	0.94 ± 0.09
C15:0	0.22 ± 0.01	0.24 ± 0.04	0.28 ± 0.06	0.29 ± 0.02	0.24 ± 0.03	0.23 ± 0.04	0.21 ± 0.03
C16:0	21.85 ± 0.37	18.82 ± 0.87	19.33 ± 1.05	17.81 ± 0.50	20.33 ± 1.07	19.63 ± 2.15	18.15 ± 1.51
NI	0.20 ± 0.04	0.23 ± 0.03	0.19 ± 0.02	0.21 ± 0.02	0.18 ± 0.04	0.18 ± 0.01	0.16 ± 0.02
C16:1*c*	3.61 ± 0.26	2.79 ± 0.27	3.18 ± 0.48	2.91 ± 0.34	3.12 ± 0.45	3.08 ± 0.75	2.68 ± 0.61
C17:0	0.22 ± 0.01	0.23 ± 0.03	0.19 ± 0.03	0.20 ± 0.01	0.22 ± 0.02	0.22 ± 0.04	0.22 ± 0.02
C17:1	0.13 ± 0.02	0.11 ± 0.02	0.10 ± 0.01	0.10 ± 0.01	0.14 ± 0.04	0.14 ± 0.07	0.13 ± 0.06
C18:0	2.97 ± 0.10	2.93 ± 0.14	2.97 ± 0.18	2.76 ± 0.08	2.96 ± 0.82	2.80 ± 0.63	2.50 ± 0.31
C18:1*t*					0.10 ± 0.02^b^	0.15 ± 0.06^ab^	0.21 ± 0.06^a^
C18:1*c* ω-9	26.32 ± 0.60	24.42 ± 0.71	24.54 ± 0.55	23.68 ± 0.41	24.70 ± 3.06	22.68 ± 2.54	20.59 ± 1.68
NI					0.19 ± 0.03^b^	0.23 ± 0.04^b^	0.34 ± 0.05^a^
C18:2*t*					0.26 ± 0.05^c^	0.44 ± 0.06^b^	0.62 ± 0.06^a^
C18:2*c* ω-6	35.89 ± 0.44^ab^	36.93 ± 2.70^a^	31.44 ± 1.59^abc^	30.46 ± 1.10^bc^	33.15 ± 3.93^abc^	30.59 ± 3.93^bc^	28.64 ± 2.49^c^
C18:3*c* ω-6	0.12 ± 0.01^c^	0.12 ± 0.01^c^	0.11 ± 0.01^c^	0.14 ± 0.01^bc^	0.14 ± 0.02^bc^	0.16 ± 0.03^ab^	0.18 ± 0.01^a^
C18:3*c* ω-3	2.56 ± 0.09^d^	7.71 ± 0.74^c^	11.91 ± 0.78^b^	16.13 ± 1.32^a^	2.28 ± 0.25^d^	1.90 ± 0.17^d^	1.54 ± 0.09^d^
CLA 9*c*11*t*					3.18 ± 0.60^c^	6.07 ± 0.89^b^	9.58 ± 0.80^a^
CLA 10*t*12*c*					0.03 ± 0.00^c^	0.07 ± 0.00^b^	0.11 ± 0.01^a^
CLA 10*c*12*c*					1.51 ± 0.22^c^	2.91 ± 0.37^b^	4.43 ± 0.49^a^
C20:2 ω-6	0.29 ± 0.01^a^	0.22 ± 0.03^b^	0.19 ± 0.02^bc^	0.16 ± 0.01^c^	0.20 ± 0.03^bc^	0.21 ± 0.03^bc^	0.17 ± 0.01^bc^
C20:3 ω-6	0.13 ± 0.02	0.12 ± 0.02	0.12 ± 0.03	0.09 ± 0.01	0.11 ± 0.01	0.10 ± 0.02	0.10 ± 0.02
C20:4 ω-6	0.84 ± 0.08^a^	0.58 ± 0.06^bc^	0.50 ± 0.07^bc^	0.42 ± 0.03^bc^	0.61 ± 0.13^b^	0.57 ± 0.16^bc^	0.39 ± 0.05^c^
C22:2					0.10 ± 0.01^b^	0.15 ± 0.03^a^	0.15 ± 0.01^a^
C 24:0	0.08 ± 0.01^c^	0.23 ± 0.02^b^	0.27 ± 0.07^ab^	0.34 ± 0.04^a^			
PA					2.13 ± 0.16^c^	3.37 ± 0.43^b^	4.99 ± 0.74^a^
α-ESA					0.19 ± 0.03^b^	0.25 ± 0.03^ab^	0.28 ± 0.05^a^
NI	0.17 ± 0.02^b^	0.34 ± 0.03^a^	0.41 ± 0.13^a^	0.34 ± 0.02^a^			
C22:6 ω-3	0.26 ± 0.03^ab^	0.33 ± 0.05^a^	0.33 ± 0.10^a^	0.24 ± 0.02^ab^	0.16 ± 0.04^bc^	0.12 ± 0.04^c^	0.06 ± 0.00^c^
**TL (%)**	74 ± 17	76 ± 10	75 ± 7	75 ± 11	71 ± 7	69 ± 15	67 ± 9

Results expressed as mean ± standard deviation (n = 4) of the fatty acid percentages; different letters in the same row are statistically different from each other (*p* <0.05)

*NI* not identified, *CLA* conjugated fatty acid (C18:2), *PA* punicic acid, α*-ESA* α-eleostearic acid, *TL* total lipids

**Table 4:** Fatty acids (FAs) profile in epididymal adipose tissue of rats supplemented with different oils

FAs (%)	Control	LNA 1 %	LNA 2 %	LNA 4 %	CLNA 1 %	CLNA 2 %	CLNA 4 %
C13:0	2.72 ± 0.31	3.12 ± 0.44	2.76 ± 0.28	2.72 ± 0.40	3.38 ± 0.39	2.80 ± 0.26	3.24 ± 0.39
C14:0	1.08 ± 0.04	0.92 ± 0.05	0.93 ± 0.10	0.85 ± 0.07	1.00 ± 0.07	0.99 ± 0.06	0.95 ± 0.11
C15:0	0.27 ± 0.02^a^	0.27 ± 0.03^a^	0.21 ± 0.02^b^	0.20 ± 0.00^b^	0.25 ± 0.04^ab^	0.23 ± 0.02^ab^	0.23 ± 0.03^ab^
C16:0	20.57 ± 0.13	18.87 ± 0.47	17.85 ± 0.92	16.01 ± 0.27	19.36 ± 1.85	19.12 ± 2.50	18.01 ± 2.17
NI	0.27 ± 0.02^a^	0.26 ± 0.02^a^	0.25 ± 0.05^a^	0.21 ± 0.02^ab^	0.15 ± 0.06^b^	0.13 ± 0.05^b^	0.15 ± 0.02^b^
C16:1*c*	3.63 ± 0.23	3.17 ± 0.23	3.34 ± 0.45	3.36 ± 0.53	3.74 ± 0.73	3.60 ± 0.85	3.44 ± 0.76
C17:0	0.24 ± 0.01	0.25 ± 0.02	0.22 ± 0.01	0.20 ± 0.01	0.23 ± 0.03	0.22 ± 0.02	0.21 ± 0.02
C18:0	2.87 ± 0.12	2.84 ± 0.07	2.78 ± 0.13	2.57 ± 0.09	2.61 ± 0.42	2.59 ± 0.39	2.38 ± 0.32
C18:1*t*					0.34 ± 0.51	0.14 ± 0.02	0.17 ± 0.04
C18:1*c* ω-9	26.02 ± 0.30^a^	24.74 ± 0.35^ab^	24.24 ± 0.54^ab^	23.26 ± 0.33^ab^	23.86 ± 1.85^ab^	22.82 ± 1.98^bc^	20.28 ± 1.78^c^
NI					0.19 ± 0.03^a^	0.27 ± 0.04^ab^	0.33 ± 0.05^b^
C18:2*t*					0.31 ± 0.05^a^	0.44 ± 0.04	0.72 ± 0.11^b^
C18:2*c* ω-6	37.05 ± 0.27^a^	35.17 ± 0.56^ab^	32.83 ± 1.38^ab^	31.09 ± 0.63^bc^	32.99 ± 3.60^a^	31.13 ± 4.21^bc^	27.89 ± 2.41^c^
C18:3*c* ω-6	0.13 ± 0.02^ab^	0.13 ± 0.01^ab^	0.09 ± 0.04^ad^	0.05 ± 0.01^cd^	0.17 ± 0.03^b^	0.19 ± 0.04^b^	0.17 ± 0.04^b^
C18:3*c* ω-3	2.75 ± 0.09^a^	8.02 ± 0.25^b^	12.11 ± 0.29^c^	17.17 ± 1.57^d^	2.54 ± 0.25^a^	2.13 ± 0.30^a^	1.89 ± 0.24^a^
CLA 9*c*11*t*					3.38 ± 0.66^a^	5.71 ± 0.78^b^	9.40 ± 1.10^c^
CLA 10*t*12*c*					0.04 ± 0.01^a^	0.07 ± 0.01^b^	0.11 ± 0.02^c^
CLA 10*c*12*c*					1.52 ± 0.21^a^	2.56 ± 0.30^b^	4.11 ± 0.52^c^
C20:2 ω-6	0.24 ± 0.02	0.25 ± 0.02	0.22 ± 0.02	0.18 ± 0.02	0.24 ± 0.04	0.23 ± 0.04	0.20 ± 0.01
C20:3 ω-6	0.14 ± 0.02^a^	0.13 ± 0.02^ab^	0.13 ± 0.02^ab^	0.12 ± 0.01^ab^	0.14 ± 0.01^a^	0.11 ± 0.02^ab^	0.10 ± 0.01^b^
C20:4 ω-6	1.14 ± 0.21^a^	0.71 ± 0.16^ab^	0.71 ± 0.08^ab^	0.61 ± 0.13^b^	0.84 ± 0.28^ab^	0.75 ± 0.25^ab^	0.49 ± 0.12^b^
C22:2					0.11 ± 0.03^a^	0.14 ± 0.02^ab^	0.16 ± 0.01
C24:0	0.10 ± 0.02^a^	0.26 ± 0.03^b^	0.34 ± 0.09^b^	0.48 ± 0.09^c^			
PA					2.13 ± 0.25^a^	3.04 ± 0.24^a^	4.83 ± 0.87^b^
α-ESA					0.18 ± 0.04^a^	0.23 ± 0.03^ab^	0.30 ± 0.09^b^
C22:4 ω-6	0.19 ± 0.03^a^	0.11 ± 0.02^b^	0.08 ± 0.01^bc^	0.05 ± 0.01^c^	0.12 ± 0.03^b^	0.09 ± 0.03^bc^	0.05 ± 0.02^c^
NI	0.15 ± 0.03^a^	0.37 ± 0.06^b^	0.49 ± 0.10^b^	0.49 ± 0.09^b^	0.13 ± 0.03^a^	0.10 ± 0.03^a^	0.06 ± 0.03^a^
C22:6 ω-3	0.29 ± 0.08^ac^	0.42 ± 0.10^ab^	0.48 ± 0.06^b^	0.37 ± 0.09^ab^	0.21 ± 0.06^c^	0.18 ± 0.07^c^	0.11 ± 0.03^d^
**TL (%)**	78 ± 6^abc^	83 ± 11^ab^	71 ± 6^bc^	67 ± 10^c^	91 ± 9^a^	71 ± 12^bc^	87 ± 10^a^

Results expressed as mean ± standard deviation (*n* = 4) of the fatty acid percentages; different letters in the same row are statistically different from each other, *p* <0.05

*NI* not identified, *PA* punicic acid, α*-ESA* α-eleostearic acid, *TL* total lipids

The results presented here agree with some other studies that have shown that CLNAs are metabolized to CLAs, and also that the isomer C18:2-9*c*11*t* was detected in the tissues (liver, kidney, heart, adipose tissue, mammary gland and intestine) of groups supplemented with PA [23, 33–35]. Consequently, many of the functions of these isomers are due to CLAs. The aforementioned authors also assessed the possibility that CLNAs can be converted to CLA by a Δ13 saturation reaction performed by a NADPH-dependent enzyme which is an enzyme that recognizes conjugated trienoic acid, or is a active enzyme in the reductive pathway of leukotriene B4. Yuan et al. [25] showed that PA was rapidly converted to 9*c*,11*t*-CLA in the plasma and various tissues of rats. They observed that neither PA nor CLA were detected immediately after treatment (time zero), but both were detected in the tissues and plasma of rats 4, 8, 12 and 24 h after treatment (each rat was fed with about 645 mg of PA). The amounts of CLA and PA in the liver and plasma of the animals were larger than in the heart, kidney and adipose tissue. In our study, Tables 3 and 4 show the presence of PA in adipose tissue; CLA was present in all three tissues and the epididymal adipose tissue presented the highest percentage. These findings suggest that the incorporation of the isomers of CLAs and CLNAs is dependent/specific on tissue. According to Reena et al. [36] the composition and organization of lipids in biological membranes are important factors that determine their fluidity. In this regard, further studies are required to assess the mechanisms of action of these specific fatty acids in animal tissues.

Although the PA present in the PSO was metabolized and incorporated into tissues in the form of CLAs, no changes were observed in the morphology of the muscle tissue (gastrocnemius) of the animals. However, it can be seen that there was a significant increase in cell diameter of the groups supplemented with 4 % and LO in all the groups supplemented with PSO when compared with the control group (Table 5). Furthermore, there was a decrease in cellularity in these groups, indicating that supplementation with PSO did not increase the amount of adipose cells, but the size of the adipocytes. The cellularity was calculated from the total weight of the epididymal adipose tissue mass, divided by the mass of the adipocytes, which was obtained from the diameter.

**Table 5:** Cell diameter and muscle oxidative stress parameters of rats supplemented with different oils

Parameters	Control	LNA 1 %	LNA 2 %	LNA 4 %	CLNA 1 %	CLNA 2 %	CLNA 4 %
Epididymal cells							
Diameter (μm)	70 ± 8.0^c^	76 ± 1.6^bc^	78 ± 9.0^bc^	84 ± 4.6^ab^	83 ± 3.3^ab^	88 ± 5.9^a^	90 ± 1.6^a^
Cellularity	7.1 × 10^-9 ab^	7.6 × 10^-9 a^	6.1 × 10^-9 abc^	5.3 × 10^-9 c^	5.3 × 10^-9 c^	4.9 × 10^-9 c^	5.7 × 10^-9 bc^
Muscle fibers							
Average area (μm^2^)	1841 ± 149	2028 ± 311	2070 ± 155	1668 ± 70	1625 ± 64	1826 ± 312	1828 ± 145
Diameter (μm)	60 ± 2.4	66 ± 6.6	65 ± 5.2	57 ± 1.5	58 ± 2.4	60 ± 4.9	63 ± 3.2
Muscle tissue							
TBARS (nmol MDA/mg)	3.2 ± 1.2	3.2 ± 0.8	2.2 ± 0.3	1.2 ± 0.6	3.0 ± 0.7	3.3 ± 0.8	2.6 ± 0.7
SOD (U/mg)	6.0 ± 0.6^a^	5.0 ± 0.6^ab^	5.5 ± 0.9^ab^	4.6 ± 0.6^b^	4.6 ± 0.6^b^	4.4 ± 0.6^b^	4.8 ± 0.8^b^
CAT (U/mg)	1.9 ± 0.5	1.7 ± 0.4	2.1 ± 0.5	1.5 ± 0.3	1.6 ± 0.3	1.8 ± 0.4	1.5 ± 0.4
GPx (U/mg)	0.07 ± 0.02	0.05 ± 0.01	0.06 ± 0.02	0.05 ± 0.02	0.06 ± 0.01	0.05 ± 0.01	0.06 ± 0.02

Results expressed as mean ± standard deviation. Different letters in the same row are statistically different from each other (*p* < 0.05)

The ability of conjugated linoleic acid (CLA) to change body composition, increase lean body mass, and reduce body fat in different species, such as mice, hamsters, rats, pigs and humans, has been widely studied [7, 37, 38]. In the present study, because the PSO supplementation led to the incorporation of CLAs in the muscle and adipose tissues, it was expected that there would be changes in the area and diameter of the muscular tissue and in the fat cell size. However, the results for these parameters showed no significant differences. Furthermore, CLNA supplementation resulted in an increase in the diameter of the epididymal adipocytes, although this did not interfere in the cellularity. This result leads us to believe that the effects were not attributed to the 9*c*11*t*-CLA isomer, which is the main form found in animal tissue supplemented with PSO. Studies have shown that each isomer of CLA has a distinct mode of action [39–41]. According to Rahman et al. [37], the supplementation of female mice with 0.5 % isolated isomers of CLAs (9*c*11*t* or 10*t*12*c*), and with 0.5 % of a mixture of these isomers for six months, showed an increase in muscle mass that was significantly greater in the groups that received 10*t*12*c*-CLA and CLA-mix group when compared to the group supplemented with 0.5 % of 9*c*11*t* and with the control diet (corn oil). In a study using human adipocyte cells Obsen et al. [41] concluded that only the 10*t*12*c* isomer-CLA decreased the synthesis of new lipids, suggesting a mechanism for anti-obesity for this isomer. Other studies have also reported that the main isomer responsible for changing effects on lipid metabolism and body composition is 10*t*12*c*-CLA [11, 38, 39, 42]. Although there is still no consensus in the literature regarding the effects of CLAs in the modulation of body composition, this result appears to be due mainly to the isomer 10*t*12*c*-CLA. This would be a possible explanation for the results found here, since the PA present in the PSO was metabolized in the studied tissues mainly as 9*c*11*t*-CLA. Then our findings according to those found by Lopes et al. [43] who investigated the effect of CLAs in the number and size of adipocytes in inguinal and retroperitoneal adipose tissue in Wistar rats. The diet was modified by adding 5.1 % of palm oil and the experimental groups were supplemented for eight weeks with isomers of CLAs as follows: 0.6 % of 9*c*11*t*; 0.6 % of 10*t*12*c*; and 1.3 % of a mixture of 9*c*11*t* and 10*t*12*c*. Supplementation with the 9*c*11*t*-CLA isomer increased the size of the adipocytes, with a consequent reduction in the number of adipocytes per unit area. According to Queiroz et al. [44], very large adipocytes, which are beyond the exhaustion of the storage capacity of fat, become more lipolytic. This can trigger an increase in the concentration of free fatty acids in plasma and also damage non-adipose organ function, which is a process identified as lipotoxicity.

The TBARS values in the muscle tissue (gastrocnemius) of the animals supplemented with oils rich in LNA and CLNA/PA did not differ between the groups. There were also no differences between the studied groups with respect to the activity of antioxidant enzymes GPx and CAT, while the activity of SOD significantly decreased in the groups LNA 4 % and all the CLNA groups (1 %, 2 % and 4 %) in comparison to the control group (Table 5). Studies have shown that CLNAs suppress tumor cell growth by a mechanism that involves lipid peroxidation [16, 45, 46]. Some researchers have also reported that conjugated fatty acids have antioxidant activity and that this could be a possible explanation for their beneficial health effects [27, 47, 48]. According to Yang et al. [34], although the mechanisms of biological activities related to CLNAs are linked to oxidation, controversial results have been reported. It has been argued that the presence of PUFAs in lipids increases susceptibility to lipid oxidation, therefore requiring an increase in the activity of antioxidant enzymes [superoxide dismutase (SOD), catalase (CAT) and glutathione peroxidase (GPx)] to reverse this situation [36]. These authors showed that rats supplemented with oil rich in unconjugated PUFAs had increased activity of antioxidant enzymes (SOD, CAT and GPx) in liver tissue when compared with the control, which received oil that was rich in saturated fatty acids. According to the results of the present study, no significant differences were observed in the lipid peroxidation of the muscle tissue of the animals supplemented with oils rich in PUFAs, or the activity of CAT and GPx enzymes that tissue.

However, the level of SOD was reduced in the gastrocnemius muscle of the groups receiving PSO when compared to the control, but there is no dose–response relationship with CLA content. It's possible that 1 % level of PSO showed maximum reducing effect in muscle SOD activity comparated to control group. Further there was no significant difference between groups treated nor a dose–response relationship in the SOD activity of the LNA groups. Other authors also found no dose–response effect of CLNAs on their works [49]. According to Reena et al. [36], a reduction in the activity of antioxidant enzymes may predispose cells to damage by free radicals. Saha et al. [49] report that the decreased SOD activity in organs suggests that the accumulation of superoxide anion radical might be responsible for increased lipid peroxidation. Santos-Zago et al. [50] studied the effects of supplementation of mice with CLAs mix (2 % in relation to the feed intake) for 42 days and observed a significant reduction in catalase activity in serum. They concluded that the reduction of catalase activity may indicate a reduced production of peroxide which, in turn, indicates a lower degree of oxidative stress. This work PSO did not affect muscle lipid peroxidation, but reducing the levels of SOD. Thus, in model of health rats, SOD could protect the tissue lipid membrane of lipid peroxidation; it is a possible explanation for the reduced levels of SOD these groups. Yuan et al. [5] reported that oxidative stress is associated with various clinical conditions and chronic diseases, and that CLNA isomers could play a role in ameliorating oxidative stress. However, given the small number of studies in the literature associated with the antioxidant activity in animal organisms and the results we obtained, (which indicated a reduction in SOD enzyme activity in the gastrocnemius muscle of animals given CLNA in three concentrations) further studies are needed to identify the action of PSO as an antioxidant in animal organisms. Saha and Gosh [49] analyzed the influence of two isomers of CLNA (0.5 % and 1 % of total lipids offered for 21 days) in antioxidant activity against oxidative stress induced by arsenic. The results showed that sodium arsenite altered the activity of antioxidant enzymes in plasma and tissue homogenates, while the pressure increased the activity of SOD, CAT and GPx to normal levels, but the results showed no dose–response effect.

It not only the amount of total calories but also the fat content or fatty acids profile dietary can make them more susceptible to free radicals interact with membrane lipids leading to the production of lipid hydroperoxides. Thus, the intra-cellular antioxidant system (SOD, CAT and GPx) play an important role under physiological conditions. Our results show that there was no change in total caloric intake, but existed a reduction in food intake in order to compensate the calories ingested by supplementation with the oils. Moreover there were no changes in muscle and adipose tissues weights, only on their composition in LNA and CFAs accumulation between treated groups. Therefore the higher LNA accumulation and the CLA-9*c*11*t* presence can explain the alterations in SOD activity and adipocytes size.

**Fig. 1:**
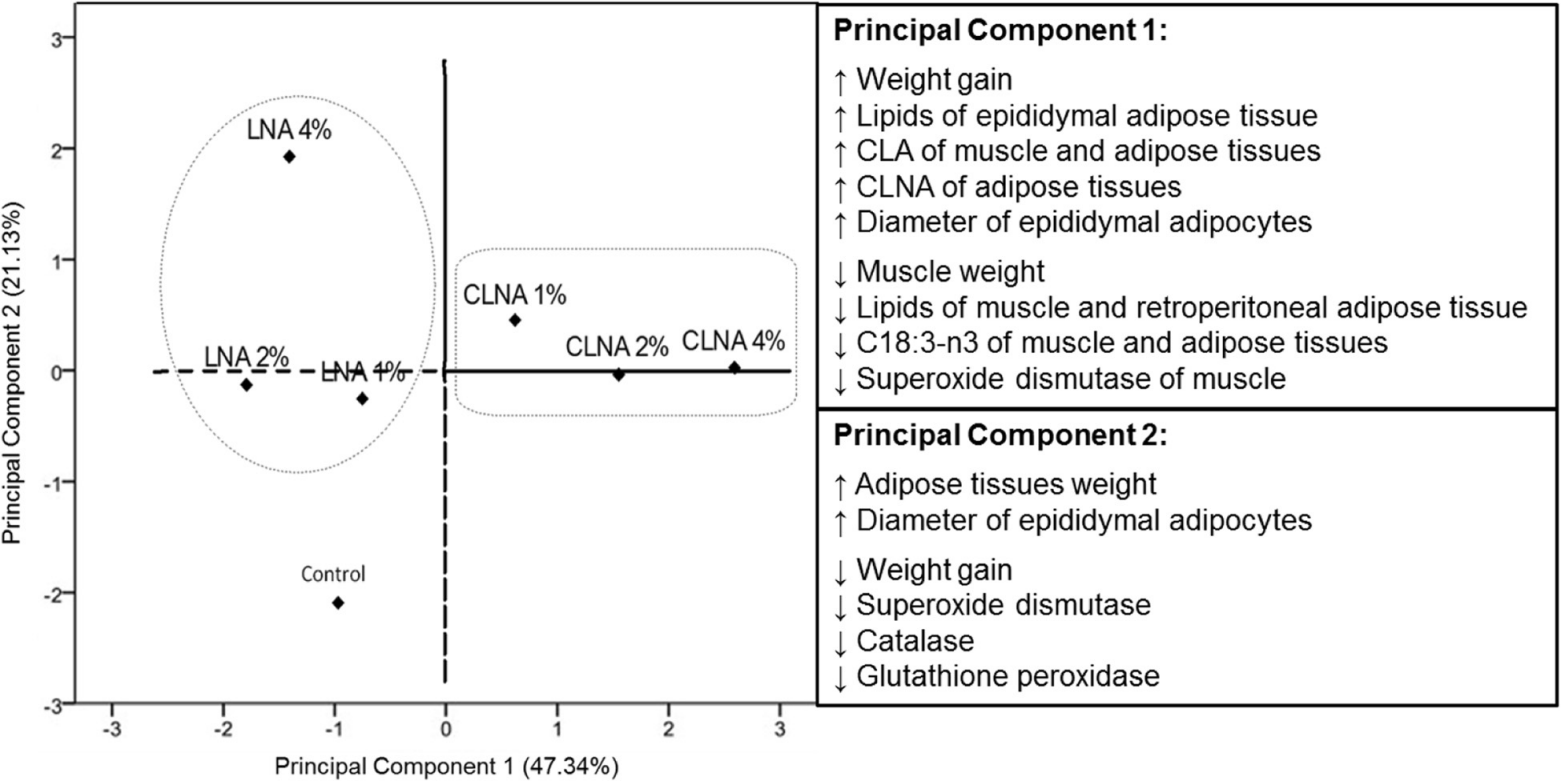
Principal component analysis (PC1 × PC2) to show differences in the evaluated parameters. The groups evaluated were grouped in different quadrants on the graph. Those who are on the positive side of the principal component 1 (x-axis) have the characteristics that are shown in the first frame of the figure. Consequently, the groups that are on the negative side of the x-axis have the opposite characteristics. This interpretation is also applied to the principal component 2 (y-axis), whose representative characteristics are show in the second frame of the figure

In order to verify the associations between the characteristics studied in the muscle and adipose tissues of the rats included in the control group, LNAs and CLNAs, principal component analysis (PCA) was applied to the data, which had been previously auto-scaled (Fig. 1). According to the figure, it can be seen that the groups were arranged separately, with a cumulative variance of 68.47 % (PC1 = 47.34 % and PC2 = 22.13 %, of the total variation); mainly the groups that received pomegranate seed oil (CLNA). PC1 was highly contributed by larger weight gain, total lipids and diameter adipocyte in epididymal adipose tissue, CLA and CLNA in the three tissues, as lower weight and superoxide dismutase activity in muscle, total lipids in muscle and retroperitoneal adipose tissue, and LNA isomer in the three tissues. PC2 was mainly correlated to larger weight of adipose tissues and diameter adipocyte, as lower weight gain and enzyme activities. The control group had negative scores to the two PC while the CLNAs groups had positive scores for both components. According to this analysis it can be noted that CLNA was linked with greater weight gain, larger adipocyte diameter and lower muscle weight. These results show that in healthy rats, supplementation with CLNA in the conditions set forth herein did not show a beneficial effect in reducing adipose tissue or increasing lean mass.

In rats under the conditions mentioned in our study, some of the beneficial effects of PSO were not been found. It seems that the main cause of this controversy is the physiological conditions of animals, because most of the rodents studies that showed significant effects of PA on body composition or antioxidants system were performed on high-fat diets, overweight and/or induced stress conditions animals. This suggests that age, species, tissue, type of supplementation and physiological conditions could to influence in effects of conjugated fatty acids. So the controversial results in our study may be due to the normal weight of our animals, the duration of supplementation, the type of conjugated isomers and supplementation without exercise training or stress condition. Controversial results also were related in researches with humans when compared to animals [51, 52]. Further researches are needed to clarify this. Despite the fact that our study had some limitations, it does not seem that these factors could have influenced our findings significantly. The major limitation was inability to measure the SOD gene expression. Our results could be better explained by an analysis of the SOD gene expression in muscle and an additional study under of other possible changes in adipose tissue.

## Conclusions

Supplementation with LO and PSO did not affect body weight gain or muscle or fat tissues. LO was able to increase the percentage of LNA in all tissues in a dose-dependent manner, while punicic acid (CLNA) was found in trace amounts and was metabolized and incorporated in the form of CLAs, mainly the isomer C18:2-9*c*11*t*. The increase in the concentration of LNA and/or CLNAs did not interfere significantly in lipid peroxidation and antioxidant enzyme activity in muscle tissue. Our findings show that PSO can be used as a source of CLAs, but that it does not cause changes in body modulation and does not interfere significantly in the antioxidant activity of healthy animals.

## Methods

Pomegranate seed oil (PSO), which was obtained by cold pressing pomegranate seeds, was purchased from Green Source Organics (Boynton Beach, USA). Samples of linseed oil (LO), which were also obtained by cold pressing, were provided by Vital Âtman (Uchôa, Brazil) and were used for comparison. The major fatty acid of LO is a non-conjugated analog of punicic acid. The principal fatty acids found in the oils (determined by GC-MS) were: 6 % C18:1-9*c*, 7 % C18:2-9*c*12*c*, 55 % C18:3-9*c*11*t*13*c* (punicic acid); 16 % C18:3-9*c*11*t*13*t* and 7 % C18:3-9*t*11*t*13*c* for PSO; and 22 % C18:1-9*c*, 15 % C18:2-9*c*12*c*, and 52 % C18:3-9*c*12*c*15*c* (LNA) for LO.

This study was approved by the Ethics Committee on Animal Experiments of the Faculty of Pharmaceutical Sciences (Craft number: CEUA/FCF/72/2008/204), University of São Paulo, Brazil. To evaluate the effect of PSO and LO *in vivo*, 56 male Wistar rats, weighing between 50 and 70 g (four weeks old), were used in the experiment. A balanced commercial diet (Nuvilab CR-1) and water were offered *ad libitum*. The animals were kept in polypropylene cages (four rats/cage) in an environment with controlled lighting (12 h light/dark cycle) at 25 °C and 60 % humidity throughout the experimental period. The animals were distributed in the following seven groups of eight animals each, according to the initial weight (so that the average weight between the groups were similar): control, rats treated with water; LNA 1 %, rats treated with 1 % LO; LNA 2 %, rats treated with 2 % LO; LNA 4 %, rats treated with 4 % LO; CLNA 1 %, rats treated with 1 % PSO; CLNA 2 %, rats treated with 2 % PSO; and CLNA 4 %: rats treated with 4 % PSO.

After a seven-day acclimatization period, the animals started receiving the supplementation oils intragastrically (po) using a gavage needle, whose percentages were compared to the average daily feed intake. The animals were weighed every week to calculate the weight gain, and the feed efficiency coefficient (FEC) was obtained according to the following relationship: animal weight gain (g) ÷ feed intake (g). The experiment was conducted for 40 days and on day 41, after fasting for eight hours, the animals were anesthetized. The gastrocnemius muscle and the adipose tissues (retroperitoneal and epididymal) were collected, weighed, and then frozen in liquid nitrogen and stored at −80 °C until analysis.

### Fatty acids profile of tissues

The lipids from the tissues were extracted using the method described by Folch et al. [53]. The fatty acids were transformed into methyl esters in accordance with the method of alkaline esterification proposed by Christie et al. [54], using NaOCH_3_ in methanol. The extract was then injected into the gas chromatograph [GC - Shimadzu 2010 with flame ionization detector and fused silica (100 m and 0.25 mm internal diameter/SP-2560) column] following the schedule described by Baublits et al. [55]. The identification of fatty acids was based on comparison with the retention times for the mixture of methyl esters of the standards C4-C24 (Sigma 18919), PUFA-2 (Sigma 47015-U), CLAs (Sigma O5632/16413) and punicic acid. The retention times were compared and identified in accordance with what has been presented in the literature. The results were expressed as mean ± standard deviation of the percentages of fatty acids present.

### Morphometry of tissues

A small piece of muscle tissue from four animals from each group was collected and immediately fixed in formalin (10 %). Sections (5 μm) were prepared and stained with hematoxylin-eosin (HE) for the photomicroscopic observations. The muscle fibers were analyzed by quantification of the area and diameter of 100 fibers, which were randomly obtained from the muscle tissue of each animal. The image acquisition was performed at 400x magnification (40x objective) using a Cool SNAP-Procf (Media Cybernetics Inc., USA) camera coupled to a microscope (Nikon Eclipse - E800, Japan). The measurements were performed using the Image-Pro Plus version 4.5 computerized imaging system (Media Cybernetics Inc.). We calculated the area and the average diameter per animal, and from these values the results were expressed as mean ± standard deviation.

A small fragment of epididymal adipose tissue was also collected from four animals in each group and the adipocytes were isolated by collagenase tissue digestion using the technique described by Rodbell [56] with some modifications [57]. The fat coxin was chopped with scissors into fine pieces and incubated in digestive buffer (DMEM, 20 mM HEPES, 4 % BSA, collagenase II - Sigma®- 1.0 mg/ml, pH = 7.45) for about 30 min at 37 °C in a water bath with orbital shaking (150 rpm). The sample was subsequently filtered through a plastic sieve with fine mesh (which retains tissue debris and undigested vessels) and washed three times with 25 ml of buffer (EHB buffer - Earle's salts, 20 mM HEPES, 1 % BSA, sodium pyruvate 1 mM without glucose, pH = 7.45) maintained at 37 °C. For the morphometric analysis, cell suspension aliquots were evaluated under an optical microscope (100x magnification) using a graduated ocular mobile diameter for cell measurement. In each preparation, assuming that the isolated adipocytes were spherical, the diameter of 100 cells was measured with the aid of the Motic Images Plus images, version 2.0 computerized system. This calculated the average diameter (μM) per animal cell and from these values, averages and final standard deviations, which were considered representative of each group.

### Muscle homogenate and protein quantification

The muscle tissue was weighed and homogenized with a potassium phosphate buffer 0.1 M (pH 7.0) at a proportion of 1:4 (m/v). The homogenates were centrifuged at 15.000 rpm for 30 min at 4 °C and the supernatant was used for the evaluation of lipid peroxidation and antioxidant enzyme activity.

The protein content in the tissue homogenates was performed in triplicate by the method of Bradford [58], obtaining readings at a wavelength of 595 nm in a UV/Vis spectrophotometer (Spectronic Genesystm® 20, Rochester, USA). The amount of protein in the samples was calculated from the standard curve using bovine serum albumin (Sigma A4503).

### Lipid peroxidation and antioxidant enzyme activity

The determination of lipid peroxidation in the muscle homogenates was carried out by measuring the production of thiobarbituric acid reactive substances (TBARS) using the method described by Ohkawa et al. [59] with minor adaptations. The TBARS concentrations were calculated using a standard curve for 1,1´,3,3´-(TEP) tetraethoxypropane (10–4 mol/L) and were expressed as μmol of malondialdehyde (MDA) per milligram of protein.

The cytoplasmic superoxide dismutase (SOD) activity was evaluated according to the methods described by [60], using a reaction medium containing cytochrome C (100 mM), xanthine (500 mM), ethylenediaminetetraacetic acid (1 mM), potassium cyanide (200 mM) and potassium phosphate buffer (0.05 M - pH 7.8). The results were expressed as units per milligram of protein. One unit of SOD activity was defined as the amount of enzyme required to inhibit the reaction rate by 50 % at 25 °C.

The catalase (CAT) activity was measured spectrophotometrically by calculating the rate of degradation of H_2_O_2_; the substrate of the enzyme was at 37 °C and pH 8.0, according to the methods described by [61]. The results were expressed as mMol of hydrogen peroxide decomposed per minute per milligram of protein.

The glutathione peroxidase (GPx) activity was determined as described by [62] by measuring the decay of the optical density at 340 nm, which was promoted by the oxidation of NADPH during the reduction of oxidized glutathione, which was catalyzed by glutathione reductase. The results were expressed as units per milligram of protein. The unit of enzyme activity was defined as the amount of enzyme required to oxidize one mol of NADPH per minute at 30 °C at pH 7.0.

### Statistical analysis

The results were expressed as mean ± standard deviation (*n* = 3). Initially, the homogeneity of variances was checked by the Hartley/Levene test and then one-way analysis of variance (ANOVA) was applied to check for differences between the groups (control, LNAs and CLNAs). Fisher’s LSD test was used to statistically determine significant differences in the means. When the data were not normal the Kruskal-Wallis test was used in order to evaluate the statistical differences between the groups. Probability values (*p*-value) below 0.05 were considered to be statistically significant [63].

In order to detect associations between all the results found in the rats included in the studied groups, principal component analysis (PCA) was used to project the groups in the factor-place (PC1 vs PC2) based on the biochemical responses assessed in the study. In this study, PCA was used to better visualize the results obtained experimentally because there were many variables involved in the response of the groups. PCA aims to reduce the dimensionality of the original data set, preserving the greatest amount of information (variance) as possible. For this purpose, the experimental results were autoscaled using the z-score [(μ - X)/σ] and the PCA analysis was based on linear correlations in which the variances were calculated as sums of squares/n-1 [64].

## Abbreviations

CAT: catalase
CFAs: conjugated fatty acids
CLA: conjugated linoleic acid
CLNA: conjugated α-linolenic acid
FEC: feed efficiency coefficient
GPx: glutathione peroxidase
LA: linoleic acid
LNA: α-linolenic acid
LO: linseed oil
PA: punicic acid
PCA: principal component analysis
PSO: pomegranate seed oil
SOD: superoxide dismutase
TBARS: thiobarbituric acid reactive substances
α-ESA: α-eleostearic acid
